# Vaccine hesitancy: understanding better to address better

**DOI:** 10.1186/s13584-016-0062-y

**Published:** 2016-02-01

**Authors:** Dewesh Kumar, Rahul Chandra, Medha Mathur, Saurabh Samdariya, Neelesh Kapoor

**Affiliations:** Department of Community Medicine and Family Medicine, All India Institute of Medical Sciences, Basni-II, Jodhpur, Rajasthan 342005 India; Department of Community Medicine, Rohilkhand Medical College and Hospital, Bareilly, U.P, 243006 India; Department of Community Medicine and Family Medicine, All India Institute of Medical Sciences, Basni-II, Jodhpur, Rajasthan 342005 India; Department of Radiation Oncology, All India Institute of Medical Sciences, Basni-II, Jodhpur, Rajasthan 342005 India; RMNCH + A Scale up project, IPE Global/USAID, Sixth Floor, DSHM, B block, Vikas Bhawan-2, Civil Lines, New Delhi, 110054 India

**Keywords:** Vaccine hesitancy, Vaccine confidence, Vaccine decision making, Vaccination

## Abstract

Vaccine hesitancy is an emerging term in the socio-medical literature which describes an approach to vaccine decision making. It recognizes that there is a continuum between full acceptance and outright refusal of some or all vaccines and challenges the previous understanding of individuals or groups, as being either anti-vaccine or pro-vaccine. The behaviours responsible for vaccine hesitancy can be related to confidence, convenience and complacency. The causes of vaccine hesitancy can be described by the epidemiological triad i.e. the complex interaction of environmental- (i.e. external), agent- (i.e. vaccine) and host (or parent)- specific factors. Vaccine hesitancy is a complex and dynamic issue; future vaccination programs need to reflect and address these context-specific factors in both their design and evaluation. Many experts are of the view that it is best to counter vaccine hesitancy at the population level. They believe that it can be done by introducing more transparency into policy decision-making before immunization programs, providing up-to-date information to the public and health providers about the rigorous procedures undertaken before introduction of new vaccines, and through diversified post-marketing surveillance of vaccine-related events.

## Introduction

Amongst all public health interventions, vaccines top the list (in efficacy) and saving millions of lives each year [1]. The success stories of eradication of small pox from the world, and the elimination of poliomyelitis from four of the World Health Organization regions, reflect highly on vaccination programs. They have immensely contributed to the decline in mortality and morbidity of many infectious diseases [2]. Success in vaccination programs is dependent on a high vaccination coverage rate. This directly protects the vaccinated individuals, and indirectly the whole community, by providing herd immunity and thereby reducing the transmission of vaccine preventable diseases (VPDs) [3].

The high rate of childhood vaccination coverage in most developed countries indicates that vaccination remains a widely accepted public health measure [4]. But the national estimates can be misleading and may not show the real picture of under-vaccinated or unvaccinated communities. Various outbreaks of VPDs including measles, poliomyelitis, diphtheria and pertussis in several parts of the developed world have mainly been linked to under-vaccinated or non-vaccinated communities [5–8]. The reasons for under-vaccination in the developing and developed world are varied and have been studied in the past. It has also been noted that many vaccinated individuals have doubts and concerns regarding vaccination [9, 10].

### Concept of vaccine hesitancy

The waning of public confidence in vaccines worldwide is a cause for concern and a major challenge for public health experts [11, 12]. The phenomenon was originally described as “vaccine resistance” or “vaccine opposition” by researchers but, lately, these expressions have been abandoned and a new term, “vaccine hesitancy” (VH) has emerged, replacing the older expressions, to describe the reluctance to be vaccinated. ***Vaccine hesitancy according to Strategic Advisory Group of Experts (SAGE) Vaccine Hesitancy working group of World Health Organization (WHO) refers to delay in acceptance or refusal of vaccines despite availability of vaccine services. Vaccine hesitancy is complex and context specific, varying across geographies and vaccine types. It is influenced by factors such as complacency, convenience and confidence***. Vaccine complacency is known to be present where the risk of vaccine preventable diseases is perceived to be low and where vaccination is not considered essential. It has been observed that vaccine hesitancy is heavily impacted by lack of confidence in the vaccine’s safety and efficacy as well as fears regarding the reliability and competence of health system. Additionally, the quality of vaccination services and their convenience (e.g. physical availability, geographical accessibility and affordability) as well as the patient’s willingness to pay, are all factors that impact the decision of whether or not to be vaccinated [13]. The term is useful for situations where vaccination services are available but vaccine acceptance is lower than the expected. Before this term was adopted and defined by the working group of SAGE-WHO, researchers used many different terminologies for this behavioral phenomenon (Table 1) [14–16].

**Table 1:** Various terminologies for vaccine related behavioural phenomenon

S.no	Researchers	Terms
1.	Gust et al. (Parental attitudes regarding vaccination)	Immunization advocates
		The go alongs to get alongs
		Health advocates
		Fence sitters
		Worried
2.	Keane et al. (Parent profiles)	Vaccine believer: parents who are convinced of the benefits of vaccination
		Cautious: parents emotionally involved with their child and who have an hard time watching them being vaccinated
		Relaxed: parents who were characterized by some scepticism about vaccines
		Unconvinced: parents who distrusted vaccinations and vaccination policy
3.	Benin et al. (Mother’s attitudes and actions)	Accepters: who agreed with or did not question vaccination
		Vaccine-hesitant: who accepted vaccination but had significant concerns about vaccinating their infants
		Late vaccinators: who purposely delayed vaccinating or chose only some vaccines
		Rejecters: who completely rejected vaccination

**Fig. 1:**
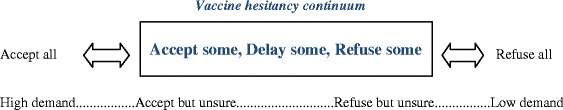
Vaccine hesitancy continuum

Attitudes towards vaccines cannot be polarized into anti-vaccine or pro-vaccine as previously thought but, rather, a continuum between full acceptance, and outright refusal, of some or all vaccines (Fig. 1) [13]. This is a complex phenomenon and vaccine specific issues must be understood contextually and conceptually [17]. It has also been determined by SAGE that although vaccine hesitancy may be present in circumstances where low vaccine uptake prevails due to flaws in vaccine availability such as stock-outs, infeasible travel/ distances to reach immunization clinics, missing vaccine program communication, or curtailment of vaccine services due to conflict, a natural disaster or other disruption, it is not always the principle driver of unvaccinated or under vaccinated members of the population. So, in low uptake situations where system failure is the major factor, hesitancy may be present but the priority is to address the factors limiting the accessibility and availability of vaccines. This means that vaccine coverage estimates cannot be regarded as a reliable indicator of vaccine hesitancy.

Research has shown that vaccination decision-making should be studied and understood in a broader socio-cultural context as vaccination is part of a “wider social world” and its decision making is highly influenced by various social factors [past experiences with health services, family histories, feelings of control, conversations with friends, etc.] [18]. Streefland and collaborators stated that “local vaccination cultures” develop from “shared beliefs about disease etiology, potency, efficacy and safety of modern medicine as well as vaccines and views related to preventive measures” alongwith “local health services experiences and vaccination settings” influence the individual decision about vaccination. It is also observed that concerns regarding child health and nutrition other than vaccination may take priority at times or has a role to play in the willingness to vaccinate [19].

The relevance of immunization in today’s context sometimes becomes questionable considering the legitimacy of science, expertise and medical authority [20]. The stress on health promotion about lifestyle and the growth of “consumerism” in health-care leading to individuals’ involvement in their own health decisions may have also contributed to some extent to vaccine hesitancy [21]. Traditionally the doctors were the sole directors of patient care but with the rise of informed patients, the decision-making concerning their health process is now shared with patients who want to be active participants and with health professionals.

### Determinants of vaccine hesitancy

**Fig. 2:**
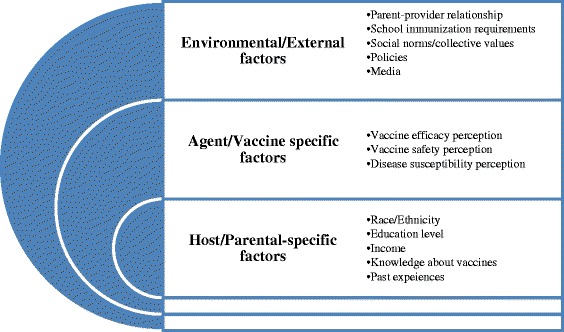
The model for understanding factors influencing parental vaccine hesitancy based on epidemiological triad: (Adopted from Gowda and Dempsey)

The factors influencing vaccine hesitancy can be explained on the basis of the epidemiological triad i.e. the complex interaction of environmental (external) factors, agent factors (vaccine) and the host (parents) specific factors(Fig. 2). The determinants of vaccine hesitancy are numerous and context specific and are presented separately but it is important to understand and acknowledge their interrelatedness [22].

### Environmental/external factors

Patient-health professional relationship: Positive interaction is the keystone in maintaining confidence regarding vaccination [23]. The personal attitude of health care providers, along with their knowledge, determines how effectively they will recommend a vaccine to their patients. It is also known from previous reviews on nurses’ practice about the influenza vaccine that there is relationship between knowledge, attitudes and vaccination practices. A review of 12 research articles concluded that a higher degree of motivation for vaccination of influenza is proportionate to the coverage of vaccination amongst nurses and the promotion of vaccination in patients. A study in Switzerland also showed that nearly 5 % of non paediatric physicians delayed or denied MMR or DPT vaccination for their own children and the reason was the concern of “immune overload” [24–26]. Another school of thought believes that vaccine hesitancy may lead to the development of certain emotional responses amongst health care providers who face it [27].

The American Academy of Pediatrics' Committee on Bioethics showed their solidarity towards families who showed their reluctance towards immunization as they were deprived of other health care facilities as well. It has been observed that some health care professionals face problems in discussing vaccine schedules and other recent advances in the field of vaccination [28]. Decision making regarding vaccination is based on trust of health professionals, government or public health institutions and their inter-relation. These relationships are of utmost importance in acceptance of the vaccines, as the public relies on their integrity, competence and faith in the government and public health authorities giving recommendations of appropriate vaccines which are effective, uncontaminated and can be administered safely [12, 29]. Benin and collaborators have proved in their research that due to lack of trust new mothers hesitated in vaccinating their children [16]. It has been noted that health professionals are the key sources of information on vaccination to those who are refusing vaccination and to vaccine hesitant patients [30]. The patient provider relationship is significant and the development of communication skills is the soul of nourishing this art. But it has also been seen that physician targeted communication intervention doesnot reduce maternal vaccine hesitancy or improve physician self-confidence so more research needs to be undertaken to explore the effective communication strategies to combat vaccine hesitancy [31].

School immunization requirements: The parents who exempt their children from school immunization programs had increased concerns over the vaccine safety and perceived less benefit from vaccines. Also there is lack of thrust from the school and education department in informing parents that if their child is not vaccinated then the chance of contracting the disease is higher in their children and can further transmit the disease in their peer groups. When parents are not provided proper information, they fail to consider the vaccine’s importance and turn into vaccine hesitant.

Social Norms /collective values: If vaccination is viewed as a social responsibility then it can prove to be a driver in improving vaccine acceptance. If people in a community make it a norm to get their kids vaccinated and it becomes a point of social appreciation, then vaccination may improve [32]. Some qualitative studies show that vaccination is considered a routine practice in societies where everyone is getting their child vaccinated [18].

Vaccine Policies and Public Health: Some countries have laws which mandate the vaccination of children for admission in schools as a part of their policies for improving vaccine coverage but such policies have always attracted a platform for debate [33]. In a population based survey of United States of America nearly 10 % of parents were found to be against compulsory vaccination as they had negative beliefs about vaccines, safety and their protective capability [34]. Communication is an important asset of public health in providing proper information to the population. In developed nations, good quality vaccine surveillance is well established but its understanding and reliability is limited amongst general population and health care providers. There have been significant problems faced by public health professionals, policy makers and patients due to false data and information regarding vaccine safety and efficacy which has paved the path in licensing of vaccines and their inclusion in universal programs. [35]

Vaccine preventable diseases declined due to the increase in vaccines which succeeded in drawing the attention of parents and health professionals on vaccine usefulness and safety [36]. VPDs are reducing due to vaccination programs hence health professionals have no first-hand knowledge of the risks of the disease. So now attention has shifted from the risk of diseases to the risk of vaccination which is why it is appropriate to state that “vaccination is victim of its own success.” [37] Some new vaccine preventable diseases are considered to be mild like chickenpox and gastroenteritis which compromises vaccine acceptability by the family [38]. It is assumed that unacceptability of vaccines to individuals is due to the manipulations by anti-vaccination groups and also irrational, emotional and ill informed attitudes and hence interventions applied to increase the vaccine uptake in the form of probabilistic information usually fails [39].

Media and Communication: Media plays a significant role in vaccine uptake and influences the community both positively and negatively. Studies have proved that negative reports from media de-motivated the community regarding vaccine uptake [40]. The burning example of pertussis immunization shows that media controversies regarding immunization lead to decreased vaccine uptake and as a result a 10 to 100 times increase in the number of cases in unimmunized countries compared to immunized countries [41]. Nowadays the very effective platform of internet is being utilized to dispense negative publicity by anti-vaccination activists [42]. As a matter of fact anti vaccination content on the World Wide Web is amply available and is disseminating rumors, myths and wrong beliefs regarding vaccines which has led to a negative impact on vaccine uptake [43]. Actually in present scenario internet is the major source of information for people. There are various sources like social network where many experiences both positive and negative are shared by individuals. Such recitals add a new dimension to the health information: usually affected by pessimism, views which are related to vaccines, potentials and vaccine preventable diseases. There are studies which have proven that the information depicted through social websites is of inconsistent quality and that the majority of them have negative ingredients [44]. As an example, the quantum of correct information was just 51 % where association between MMR and Autism was searched by patients [45].

The most common propaganda on anti vaccination websites is regarding the “Hot lots” in vaccines, suspicion of poison in vaccines and many bad personal experiences after taking vaccines [46]. All these arguments indicate towards ‘Denialism’ by anti vaccination activists. The term Denialism has been defined as “the employment of rhetorical arguments to give the appearance of legitimate debate where there is none, an approach that has the ultimate goal of rejecting a proposition on which a scientific consensus exists.” Diethelm and McKee have proved that denialists have many tactics to prove the relation between autism and vaccination like using “Conspiracy theories”, creating fake experts, selecting those evidences which support the false results and building a bad report of vaccines in the community [47]. It has been seen that those individuals who deny or delay the vaccines are the ones who have done extensive internet searches on the vaccine related matter [48]. A very interesting study by Betsch and collaborators has concluded that anti vaccination surfing for nearly 5–10 min had influenced people’s decision of vaccinating their children in a negative manner [49].

### Agent/vaccine specific factors

Vaccine efficacy perception: Perceptions about vaccine efficacy are an integral factor in vaccine decision making for vaccine hesitant parents. There is a significant concern over the relative efficacy of vaccine induced immunity versus immunity obtained through the natural course of events with a few parents preferring immunity acquired naturally to that acquired via vaccination. People in a few parts of the world have also started raising questions over vaccines such as the Oral Polio Vaccine, that despite giving multiple doses of vaccines on National Immunization Days(NIDs) and Sub-National Immunization days(SNIDs) with good coverage and quality maintenance, countries like India still took more than one and half decades to eliminate poliomyelitis from their country. Maintaining confidence over vaccines when used for long time in the same children is a tough fight for the program managers involved in immunization programs who have to convince the community and vaccine hesitant people about their prolonged use.

Vaccine safety perception: It is a well known fact that parents hesitating for a vaccine are more concerned about the immediate side effects or adverse events due to a vaccine, but the hesitancy spectrum extends to long lasting complications including neurologic conditions as well. Additional concerns regarding vaccine safety are the number and timing of recommended vaccines. Recently, many new vaccines have been introduced and additional new vaccines are in the pipeline which will be included in the recommended vaccination schedule and this number is likely to grow in the future. This has alarmed parents about the overloading of the immune system by receiving too many antigens in a short span of time which may be harmful instead of doing good to their children. Some parents are specifically worried about the cumulative pain and discomfort faced by the children after multiple shots given at once.

Vaccination has always been the subject of many controversies which have affected vaccine acceptance of various vaccines to varying degrees in the past as well as in the present. The incidences of the controversies are often within a particular context such as the association between the hepatitis B vaccine and multiple sclerosis in France that resulted in the suspension of the universal vaccination program in the 1990s, despite the lack of substantial evidence of such an association [50]. In India the controversy arose with the introduction of pentavalent vaccine regarding its adverse effects and efficacy and thereby the rationale in its introduction was questioned [51]. The well-known vaccination scare that occurred in the United Kingdom was the false association between the MMR vaccination and autism, which rapidly spread worldwide and the concern of autism due to vaccines among parents is still present, although the purported association has been scientifically disproven [22].

Disease susceptibility perception: The perceptions of the importance of vaccination in maintaining health is an important factor for accepting vaccines. Vaccine acceptance has been found higher in those who perceive vaccination as an important entity to counter the detrimental consequences of vaccine preventable diseases. The overwhelming success of vaccination efforts has drastically reduced the incidence of VPDs all over the world, decreasing the exposure of these VPDs and their complications. This has resulted in perceiving such illnesses to be insignificant health threats. Personal experience with a limited form of a disease may have created a belief that disease related risks are low. This holds true for the varicella vaccine as many parents recall having had chicken pox in their childhood without any complications. Similarly some parents prefer their children acquiring natural immunity to giving a measles containing vaccine. Studies have proven that parents’ beliefs regarding disease susceptibility play a significant role in deciding whether their children should get vaccinated or not. Common views regarding the reasons for vaccine hesitancy are: the inclination towards natural immunity, the age old belief that occurrence of vaccine-preventable diseases leads to the development of natural immunity and the belief that better hygiene can prevent diseases, rendering vaccines unnecessary [52]. Vaccine doubts among vaccine hesitant parents are further fuelled by the synergistic imbalance created between decreasing levels of perceived disease susceptibility and increasing concerns about vaccine safety.

### Host/parental specific factors

Race, education and income: These individual characteristics may have a direct impact on the person’s concept of the risks and benefits of vaccination along with the risks and sequelae of a VPD. Some studies demonstrate that African-Americans have lower immunization coverage levels compared to other race groups in America. This supports the fact that ethnicity/race is associated with differential levels and types of immunization concerns. However recent data after adjusting for poverty status have not shown significant difference in coverage levels by racial groups [53]. One of the factors implicated in vaccine hesitancy is the level of parental education and studies in the past have demonstrated greater distrust for medical professionals amongst communities with less formal education. Due to the lower education level, their information about vaccines and their effect is less as compared to more educated parents and the parents seek out alternative sources such as family members and other parents in the community or the media for reliable information.

The propaganda of anti-vaccination messages is more than the pro-vaccination messages in these outlets contributing further to parental vaccine hesitancy. Socioeconomic factors appear to have conflicting associations with parental immunization acceptance reflecting differences in beliefs about vaccines by socioeconomic strata. In some studies parents with lower socio-economic class have shown more concern about the safety and necessity of vaccines as compared to those with higher socio-economic status [34]. In another study, parents in a higher income group were more concerned approximately two fold with the safety of the shots. The apparent contradiction could be related to differing perceptions of what vaccine safety means among the different strata of population. For example parents in high income groups may relate vaccine safety to concerns such as autism or long term neurological conditions. The influence of social factors on vaccine hesitant behaviour may be the opposite to what is assumed i.e. it is often the non-elite or minority communities that have better acceptance and higher vaccine coverage than affluent and wealthier sections of the community [54]. Hence there are other factors which highly influence the vaccine uptake are previous experiences, accessibility and convenience of vaccination.

Parent’s decision, knowledge and past experiences: There is enough evidence available which shows that parents decide for their children vaccines such as HPV, pneumococcal, seasonal flu or pandemic flu etc. Therefore the studies usually focus on parents for obtaining the information on the vaccine uptake in the community because most of these vaccines are targeted at children and adolescents. It has been observed that parents’ decision making is influenced by social factors, cultural issues and the personal experiences of the individuals [55]. Acceptance of vaccination is found to be directly proportional to the quality of services available. In the case of children, fear of needle, pain and previous bitter experiences regarding vaccination leads to vaccine hesitancy [53]. Other than the above mentioned factors vaccine rejection is also associated with strong religious beliefs along with conventional trust of natural and artificial medicines. Even in developed countries like USA, vaccine refusal is sometimes connected to religious intentions [29]. Another important determinant of sub-optimal vaccine uptake is the direct and indirect cost of vaccines which influences the parent’s decision directly and adds to vaccine hesitancy [56] Vaccination acceptance depends on individuals’ knowledge, information and awareness of when, where and who should be vaccinated. The immunization information needs to be disseminated properly to increase the knowledge of parents which will enormously aid the reduction of vaccine hesitancy.

### Way forward

Although it is quite difficult to quantify accurately the proportion of the population that could be categorized as vaccine-hesitant, there has been a growing consensus among experts worldwide that there is an increasing trend toward vaccine hesitancy. As depicted in this paper, individual decision-making regarding vaccination is a complex process and is dependent on emotional, cultural, social, spiritual and political factors as well as cognitive factors. Factually vaccine hesitancy was present even when the first vaccines were made available. However, vaccine hesitancy may have heightened by the current “changing scientific, cultural, medico-legal and media environments” despite increasing awareness about vaccines [19].

The renewed and growing interest in vaccine hesitancy has led to the development of different tools and strategies which can help to enhance vaccination acceptance which includes some social and commercial marketing principles and practices [57]. Many experts are of the view that its best to counter vaccine hesitancy at the population level and it can be done by including transparency in policy-making decisions regarding immunization programs, providing updated information to the public and health providers about the rigorous process undertaken before the introduction of new vaccines for the general population and diversified post-marketing surveillance of vaccine-related events. In addition, a special focus should be placed on listening to concerns and understanding the perceptions of the public to inform risk communication and to incorporate public perspectives in planning vaccine policies and programs.

To counter vaccine hesitancy, program managers initially must adequately identify the target population and understand the true nature of their particular vaccine and/or vaccination concerns. Then intervention strategies should be planned effectively considering the locally relevant factors operating in the population. But it is also important to bear in mind that low vaccine uptake may not be due to vaccine hesitancy alone. Finally, due to their critical role in sustaining the success of vaccination programs, there is an urgent need to undertake further research so as to understand why some health professionals, trained in medical sciences, still have doubts regarding the safety and effectiveness of vaccination. It is also worthwhile to note that causes of vaccine hesitancy vary from country to country and hence there is a need to identify locally relevant and context specific causal factors before intervention strategies to address them can be planned effectively. Although there is an effort going on at a global level to measure Vaccine Confidence Index and the insights generated will definitely help in strengthening of local and global vaccine confidence in the years to come but measuring vaccine confidence is an emerging science and a lot more needs to be done in this field [12].
